# Improved tomographic reconstruction of large-scale real-world data by filter optimization

**DOI:** 10.1186/s40679-016-0033-y

**Published:** 2016-12-03

**Authors:** Daniël M. Pelt, Vincent De Andrade

**Affiliations:** 1 Lawrence Berkeley National Laboratory, 1 Cyclotron Road, Berkeley, CA 94720 USA; 2 Computational Imaging Group, Centrum Wiskunde & Informatica, Science Park 123, 1098 XG Amsterdam, The Netherlands; 3 Advanced Photon Source, Argonne National Laboratory, 9700 South Cass Avenue, Lemont, IL 60439 USA

**Keywords:** Filtered backprojection, Gridrec, Iterative reconstruction

## Abstract

In advanced tomographic experiments, large detector sizes and large numbers of acquired datasets can make it difficult to process the data in a reasonable time. At the same time, the acquired projections are often limited in some way, for example having a low number of projections or a low signal-to-noise ratio. Direct analytical reconstruction methods are able to produce reconstructions in very little time, even for large-scale data, but the quality of these reconstructions can be insufficient for further analysis in cases with limited data. Iterative reconstruction methods typically produce more accurate reconstructions, but take significantly more time to compute, which limits their usefulness in practice. In this paper, we present the application of the SIRT-FBP method to large-scale real-world tomographic data. The SIRT-FBP method is able to accurately approximate the simultaneous iterative reconstruction technique (SIRT) method by the computationally efficient filtered backprojection (FBP) method, using precomputed experiment-specific filters. We specifically focus on the many implementation details that are important for application on large-scale real-world data, and give solutions to common problems that occur with experimental data. We show that SIRT-FBP filters can be computed in reasonable time, even for large problem sizes, and that precomputed filters can be reused for future experiments. Reconstruction results are given for three different experiments, and are compared with results of popular existing methods. The results show that the SIRT-FBP method is able to accurately approximate iterative reconstructions of experimental data. Furthermore, they show that, in practice, the SIRT-FBP method can produce more accurate reconstructions than standard direct analytical reconstructions with popular filters, without increasing the required computation time.

## Background

Advanced experimental facilities such as synchrotrons enable users to routinely perform large-scale tomography experiments, in which large amounts of data are produced in short time. In a typical experiment at a synchrotron facility, several thousand projection images, each with several thousand rows and columns of pixels, are acquired in the order of minutes. After acquisition, the data have to be processed to obtain results that are relevant to the specific research question. The entire processing pipeline usually consists of many different steps, from loading the data and pre-processing to post-processing and final image analysis. To enable direct feedback and optimization of experimental parameters, it is important that the acquired projections can be processed in a time comparable to data acquisition. This is especially important for experiments involving dynamic objects [1] or in situ experiments [2]. One of the most important steps of the pipeline is the *reconstruction* of the projection data, in which the pre-processed projections are transformed to a three-dimensional reconstructed image of the scanned object. Reconstruction usually has a large influence on the quality of the final results and is often the most time-consuming step of the entire pipeline. For large-scale tomographic data, the reconstruction step has two competing goals: it has to produce high-quality reconstructed images, but it should be computationally efficient as well.

Tomographic reconstruction is an example of an *inverse problem* in which an unknown image is reconstructed from its measured projections. In most applications of tomography, the inverse problem that has to be solved is *ill-posed* [3], which can make it difficult to find accurate reconstructions in practice. In ill-posed reconstruction problems, the amount of acquired data is not sufficient to guarantee the existence of a unique solution, and there can be infinitely many solutions that fit with the data. Furthermore, solutions to ill-posed problems are typically very sensitive to small amounts of noise in the acquired data. Due to the large number of applications for tomography, however, there has been extensive research into the topic of computing accurate tomographic reconstructions [3, 4], resulting in a wide range of different reconstruction methods.

The most popular reconstruction methods for large-scale tomography data are *direct* methods (often called *analytical* methods as well), which include the filtered backprojection (FBP) method [4] and the gridrec method [5]. Direct methods are typically computationally efficient, which make them well-suited for processing large-scale data. The approach is based on inverting a continuous model of the tomographic experiment, which assumes that an infinite number of noise-free projections are available. If the number of projections is sufficiently large, and the signal-to-noise ratio sufficiently high, reconstructions computed by direct methods are usually of high enough quality to make further analysis of the data possible. In many cases, however, it is not possible or not desirable to obtain a large number of projections with a high signal-to-noise ratio. In experiments involving dynamically changing objects, for example, the rate of acquisition has to match the rate of change in the sample [6]. Another example is experiments involving dose-sensitive objects, such as biological samples, in which the total amount of deposited X-ray dose can limit the number of projections that can be acquired [7]. Finally, parts of the experimental setup can block the beam [8], which limits the angular range for which projections can be acquired. In these cases, reconstructions computed by direct methods often contain significant artifacts, making further analysis difficult or impossible.

For tomographic reconstruction problems with limited data, *iterative* reconstruction methods are often able to produce reconstructions with higher quality than direct methods [9]. Iterative reconstruction methods are based on modeling the experiment as a linear system of equations, which is solved by an iterative optimization method. The model only assumes that data are available for the projections that are actually acquired, which can improve reconstruction quality for problems with a limited number of projections compared with direct methods. Furthermore, the problem of noise in linear systems has been studied extensively in the past, and several approaches are available to minimize the effect of low signal-to-noise ratios on the reconstructed image. Recently, much research has focused on *regularized* iterative reconstruction, in which additional regularizing terms are added to the error function that is minimized, exploiting prior knowledge about the scanned object to obtain accurate reconstructions from highly limited data. For example, if it is possible to represent the scanned object sparsely in a certain basis, compressed sensing algorithms can often be used to obtain accurate reconstructions [10]. An example of this approach is Total Variation minimization, which can be used to accurately reconstruct objects that have a sparse gradient in a wide variety of applications [11, 12]. A different approach is taken in *discrete* tomography, in which objects that consist of a limited number of materials can be accurately reconstructed by exploiting prior knowledge about the expected materials [13]. An important disadvantage of iterative reconstruction methods is their computational costs, which are typically significantly higher than those of direct methods. Regularized iterative methods usually require even higher computational costs, since they are based on extending standard iterative methods. For large-scale tomographic data, it is often the case that iterative methods are so time-consuming that they are unusable in practice, which is one of the reasons that direct methods remain popular [14].

In the past, much research has been focused on reducing the computation time required for iterative reconstruction methods. For example, tomographic operations are well suited for graphic processing units (GPUs) [15], enabling significant time reduction by exploiting them in iterative methods [16, 17]. Even when using GPUs, however, it takes hours to compute full three-dimensional iterative reconstructions for typical data sizes in modern large-scale tomographic experiments [18]. A different approach is to use massively parallelized methods on large supercomputers to improve computation time. Using 32K supercomputer nodes, for example, the computation time for a full iterative reconstruction can be reduced to minutes [19]. Supercomputing facilities, however, are expensive to build and maintain, not always available to every user, and usually have to be shared with others. Computing time might not be readily available at all times, especially during an experiment, when it is important that the acquired data can be reconstructed immediately.

A different approach has been proposed in recent works: instead of speeding up iterative methods, this approach modifies existing direct algorithms to improve the quality of the reconstructions they compute. Often, the modifications are based on changing the filtering step that is common in direct methods. In [20] for example, filters are computed by solving linear systems that are similar to the ones solved in iterative reconstruction methods. Reconstructions computed by the resulting method are very similar to reconstructions computed by iterative methods, but still require significantly more computation time compared with direct methods. In [21], a filter is proposed that is based on the popular iterative *simultaneous iterative reconstruction technique* (SIRT) method, which is extended with ray-by-ray noise weighting in [22]. During derivation of the filters, however, it is assumed that enough projections are available such that a certain approximation is accurate, and that projections are available for the full 180$^{\circ }$ range. Another example is the method proposed in [23], where filters are computed that accurately approximate the iterative SIRT method. During computation of these filters, a large number of iterative SIRT reconstructions have to be computed, which can take a prohibitively long time for problem sizes that are common in practice.

In this paper, we focus on the recently proposed SIRT-FBP method [24]. The SIRT-FBP method improves on existing filter-based methods in several important aspects. Reconstructions of the SIRT-FBP method are identical to existing direct methods except for the filtering step, resulting in computationally efficient reconstruction and enabling the use of existing highly efficient implementations. Furthermore, no assumptions on the availability of projections are made during derivation of the filter, making it applicable to problems with a limited number of projections and/or a limited angular range. Finally, computing a SIRT-FBP filter requires a computation time that is similar to a single iterative SIRT reconstruction, and filters can be precomputed and reused on problems with identical experimental setups.

In general, most literature about filter-based reconstruction methods, including [24], use small-scale simulated tomography problems to investigate the performance of their methods. Many problems can occur when applying mathematical algorithms to real-world data, however, which typically require modifications of the algorithms to solve. In this paper, we present the application of the SIRT-FBP method to large-scale real-world tomography experiments. We will focus specifically on the implementation details that are important for real-world application of the method. We begin by explaining the problem of tomographic reconstruction and popular reconstruction methods in the “Notation and concepts” section. In the “Methods” section, we give a brief explanation of the SIRT-FBP method. A more in-depth explanation of the implementation details that are important when applying SIRT-FBP to large-scale real-world data is given in the “Implementation details” section. In the “Results and discussion” section, three examples of applications of the SIRT-FBP method in practice are given, comparing reconstruction results of SIRT-FBP with several popular methods. Finally, we conclude the paper in the “Conclusions” section with some final remarks.

## Notation and concepts

### Problem definition

In this paper, we will focus on three-dimensional parallel-beam tomographic reconstruction problems. Since each slice of the three-dimensional object can be reconstructed independently from the other slices, we can model the problem as a collection of two-dimensional problems. Specifically, we model the unknown object as a two-dimensional function $f{:}\,\, {\mathbb {R}}^2 \rightarrow {\mathbb {R}}$, with the measurements $P{:}\,\, {\mathbb {R}}^2 \rightarrow {\mathbb {R}}$ defined as

1 $$\begin{aligned} P(t, \theta ) = \int _{-\infty }^{\infty } \int _{-\infty }^{\infty } f(x, y) \delta (x \cos \theta + y \sin \theta - t) {\mathrm {d}}x {\mathrm {d}}y, \end{aligned}$$

where $\delta$ is the Dirac delta function. In other words, each measurement $P(t, \theta )$ is defined as the line integral of *f*(*x*, *y*) over the line defined by $t = x \cos \theta + y \sin \theta$. The problem of tomographic reconstruction is to find the unknown image *f*, given its *projections* *P*.

In practice, only a finite number of measurements can be acquired. If measurements are acquired for $N_{\theta }$ angles and $N_d$ measurements per angle, the acquired projections are typically written as a vector ${\mathbf {p}} \in {\mathbb {R}}^{N_{\theta } N_d}$. Similarly, the unknown object is reconstructed on a $N \times N$ pixel grid, with the reconstructed values written as a vector ${\mathbf {x}} \in {\mathbb {R}}^{N^2}$. Using these definitions, we can define the tomographic experiment as the following linear system:

2 $$\begin{aligned} {\mathbf {W}} {\mathbf {x}} = {\mathbf {p}}. \end{aligned}$$

Here, ${\mathbf {W}} \in {\mathbb {R}}^{N_{\theta } N_d \times N^2}$ is called the *system matrix*, with element $w_{ij}$ equal to the contribution of pixel *j* to the measurement *i*. Since the system matrix can be extremely large for typical problem sizes in practice, the matrix is usually not computed explicitly. Instead, each multiplication of a vector with ${\mathbf {W}}$ or ${\mathbf {W}}^{\rm T}$ is computed on-the-fly [17]. In this context, multiplication with ${\mathbf {W}}$ is called *forward projection*, and multiplication with ${\mathbf {W}}^{\rm T}$ is called *backprojection*.

Several algorithms have been proposed to solve tomographic reconstruction problems. In practice, two different approaches are commonly used: *direct* methods and *iterative* methods.

### Direct methods

Direct methods are based on taking the continuous model of tomographic experiments [Eq. (1)] and finding an inversion equation for it. This continuous inversion equation is discretized afterwards, resulting in an algorithm for discrete projection data. For two-dimensional parallel-beam problems, the direct approach results in the popular FBP method. The FBP method consists of a filtering step, where the acquired projections are convolved with a filter, and a backprojection step afterwards:

3 $$\begin{aligned} {\text {FBP}}({\mathbf {p}}, {\mathbf {h}}) = {\mathbf {W}}^{\rm T} {\mathbf {C}}_{{\mathbf {h}}} {\mathbf {p}}. \end{aligned}$$

Here, ${\mathbf {C}}_{{\mathbf {h}}}$ is the convolution operator that convolves the measurements of each projection angle with the corresponding filter in ${\mathbf {h}} \in {\mathbb {R}}^{N_{\theta } N_f}$. Note that the data of each projection angle are filtered independently from the data of other projection angles. Therefore, the chosen filter can be different for each projection angle, which we will call *angle-dependent* filtering in this paper. Typically, however, a single filter ${\mathbf {h}}' \in {\mathbb {R}}^{N_f}$ is used for every projection angle. For example, a popular filter is the Ram-Lak filter, which is defined as ${\mathscr {F}} {\mathbf {h}}' = |\omega |$, where ${\mathscr {F}}$ is the Fourier transform operator and $\omega$ the Fourier frequency. Several other filters are used in practice [25], such as the Shepp–Logan, Hann, and Parzen filters. Most filters are modifications of the Ram-Lak filter, aiming to improve reconstruction results for problems with low signal-to-noise ratios and/or limited numbers of projections.

In parallel-beam geometries, FBP reconstructions can also be computed by first backprojecting the acquired data, and performing a two-dimensional filtering operation afterwards:

4 $$\begin{aligned} {\text {FBP}}'({\mathbf {p}}, {\mathbf {g}}) = {\mathbf {C}}'_{{\mathbf {g}}} {\mathbf {W}}^{\rm T} {\mathbf {p}}. \end{aligned}$$

Here, ${\mathbf {C}}'_{{\mathbf {g}}}$ is a two-dimensional convolution operator with filter ${\mathbf {g}} \in {\mathbb {R}}^{N_f \times N_f}$. If ${\mathbf {W}}{\mathbf {g}} = {\mathbf {h}}$, both Eqs. (3) and (4) will produce similar reconstruction results. We will use this equivalence during the derivation of the SIRT-FBP method in the “Methods” section.

### Iterative methods

Iterative reconstruction methods are based on a discrete model of the tomographic experiment, which can be written as a linear system [Eq. (2)]. Specifically, iterative methods compute reconstructions by solving the linear system iteratively, reducing the *projection error* in some vector norm in each iteration. In the case of the $\ell _2$-norm, i.e., $\Vert {\mathbf {y}} \Vert _2^2 = \sum _{i=0}^{n-1} y_i^2$ for ${\mathbf {y}} \in {\mathbb {R}}^n$, iterative methods compute the following reconstructions:

5 $$\begin{aligned} {\mathbf {x}}_{\mathrm {iter}}=\underset{\mathbf {x}}{{\text {argmin}}} \Vert {\mathbf {p}} - {\mathbf {W}} {\mathbf {x}}\Vert _{2}^{2}. \end{aligned}$$

By using different iterative optimization algorithms, different iterative reconstruction methods can be defined.

A popular iterative method is the SIRT method [4], which can be viewed as an application of Landweber iteration [26] to the linear system of tomography. Starting with an initial image ${\mathbf {x}}^0$, SIRT reconstructions are computed by the following iterations:

6 $$\begin{aligned} {\mathbf {x}}^{i+1} = {\mathbf {x}}^{i}+\alpha {\mathbf {W}}^{\rm T}\left( {\mathbf {p}} - {\mathbf {W}} {\mathbf {x}}^{i}\right). \end{aligned}$$

Here, $\alpha$ is a relaxation factor that influences the convergence rate and should satisfy $0< \alpha < \frac{2}{\sigma ^2}$, with $\sigma$ equal to the largest singular value of ${\mathbf {W}}$. Various ways of choosing a suitable relaxation factor are available, for example by comparing SIRT with other methods [27] or adjusting the factor at each iteration [28]. In the rest of this paper, we use $\alpha =\left( N_{\theta } N_d\right) ^{-1}$, which is related to Cimmino’s method [29] and a reasonable choice for most problems.

As explained in the “Background” section, the continuous inversion equations that are the basis of direct methods assume that projection data are available for an infinite number of noise-free measurements, which is infeasible in practice. For problems with a limited number of projections and/or a low signal-to-noise ratio, the reconstruction quality of direct methods is often insufficiently high to make further analysis possible. In these cases, iterative methods tend to produce reconstructions with less artifacts than direct methods. One reason for this is that iterative methods are based on a model that only uses projections that were actually acquired. Furthermore, by choosing a certain number of iterations or using additional regularization, the effect of noise in iterative methods can be minimized. A major disadvantage of iterative methods is their computational cost: typically, several hundreds of forward projections and backprojections have to be computed for a single reconstruction, compared with a single backprojection for filtered backprojection.

## Methods

In this section, we will introduce the SIRT-FBP method by briefly explaining the derivation of the method as published in [24].

The SIRT-FBP method approximates the iterative SIRT method by standard FBP with a specific filter. Here, we give a short introduction to the method; for a more in-depth mathematical derivation, we refer to [24]. We begin by analyzing the standard equation of the SIRT method [4] in its Landweber iteration form [Eq. (6)]. We can rewrite Eq. (6) to a matrix form by grouping the terms related to ${\mathbf {x}}$ and ${\mathbf {p}}$, resulting in the following equation:

7 $$\begin{aligned} {\mathbf {x}}^{i+1}=\left( {\mathbf {I}}-\alpha {\mathbf {W}}^{\rm T}{\mathbf {W}}\right) {\mathbf {x}}^{i} + \alpha {\mathbf {W}}^{\rm T}{\mathbf {p}} \end{aligned}$$

Note that Eq. (7) is a recurrence relation of the form ${\mathbf {z}}^{i+1} = {\mathbf {A}} {\mathbf {z}}^i + {\mathbf {b}}$, for which we can find a direct equation for the result of *n* iterations:

8 $$\begin{aligned} {\mathbf {z}}^n = {\mathbf {A}} {\mathbf {z}}^0 + \left[ \sum _{k=0}^{n-1} {\mathbf {A}}^k \right] {\mathbf {b}} \end{aligned}$$

By defining ${\mathbf {A}} = {\mathbf {I}} - \alpha {\mathbf {W}}^{\rm T}{\mathbf {W}}$ and substituting ${\mathbf {b}} = \alpha {\mathbf {W}}^{\rm T} {\mathbf {p}}$ and ${\mathbf {z}}^i = {\mathbf {x}}^i$, we can find a direct equation for the reconstruction result of *n* iterations of SIRT:

9 $$\begin{aligned} {\mathbf {x}}^n = {\mathbf {A}} {\mathbf {x}}^0 + \alpha \left[ \sum _{k=0}^{n-1} {\mathbf {A}}^k \right] {\mathbf {W}}^{\rm T} {\mathbf {p}}. \end{aligned}$$

In many cases, the initial image ${\mathbf {x}}^0$ is taken to be the zero image, in which case we can ignore the first term of Eq. (9), resulting in

10 $$\begin{aligned} {\mathbf {x}}^n = \alpha \left[ \sum _{k=0}^{n-1} {\mathbf {A}}^k \right] {\mathbf {W}}^{\rm T} {\mathbf {p}}. \end{aligned}$$

A comparison of Eq. (10) to the backproject-then-filter form of FBP [Eq. (4)] suggests that we can approximate Eq. (10) by FBP with a certain filter ${\mathbf {q}}_n$:

11 $$\begin{aligned} {\mathbf {x}}^n \approx \alpha {\mathbf {C}}_{{\mathbf {q}}_n} {\mathbf {W}}^{\rm T} {\mathbf {p}}. \end{aligned}$$

The question remains how to choose ${\mathbf {q}}_n$, such that ${\mathbf {C}}_{{\mathbf {q}}_n} \approx \sum _{k=0}^{n-1} {\mathbf {A}}^k$. It turns out that a good approximating filter can be computed by taking the *impulse response* of $\sum _{k=0}^{n-1} {\mathbf {A}}^k$:

12 $$\begin{aligned} {\mathbf {q}}_n = \sum _{k=0}^{n-1} {\mathbf {A}}^k {\mathbf {e}}_c , \end{aligned}$$

where ${\mathbf {e}}_c \in {\mathbb {R}}^{N_d}$ is a vector with the central element equal to 1, and the other elements equal to 0. In other words, we can compute a filter by starting with an image with the central pixel set to 1 and the other pixels set to 0, and iteratively apply ${\mathbf {A}} = {\mathbf {I}} - \alpha {\mathbf {W}}^{\rm T}{\mathbf {W}}$ to it, summing each resulting image. The result is an image ${\mathbf {q}}_n$ which can be interpreted as a 2D convolution filter.

Since, for parallel-beam geometries, backprojecting a sinogram and filtering afterwards is identical to filtering a sinogram and backprojecting the result, we can compute filters for standard filter-then-backproject FBP by forward projecting ${\mathbf {q}}_n$:

13 $$\begin{aligned} {\mathbf {u}}_n = \alpha {\mathbf {W}} {\mathbf {q}}_n. \end{aligned}$$

By using ${\mathbf {u}}_n$ as an angle-dependent filter in the FBP method, an approximation to the SIRT method is obtained:

14 $$\begin{aligned} {\mathbf {x}}^n \approx {\text {FBP}}({\mathbf {p}},{\mathbf {u}}_n) = {\mathbf {W}}^{\rm T} {\mathbf {C}}_{{\mathbf {u}}_n} {\mathbf {p}}. \end{aligned}$$

For more information about the mathematical derivation of the SIRT-FBP method and for a comparison between reconstruction result of standard FBP, SIRT, and SIRT-FBP for simulated phantom data, we refer to [24]. In the rest of this paper, we will focus on applying the SIRT-FBP method to large-scale real-world tomographic data.

## Implementation details

As with many tomographic reconstruction algorithms, several practical issues arise when applying the SIRT-FBP method on real-world tomographic data. In this section, we discuss implementation details that are important for real-world application of SIRT-FBP. To demonstrate the impact of some of these details, we give reconstruction results for simulated data in the “Results and discussion” section.

### Shift-invariance of the system matrix

The quality of the approximation in Eq. (14) depends on how well the $\sum _{k=0}^{n-1} {\mathbf {A}}^k$ operator is approximated by the convolution operation ${\mathbf {C}}_{{\mathbf {q}}_n}$. Note that the convolution operation is *shift-invariant*, while the $\sum _{k=0}^{n-1} {\mathbf {A}}^k$ operation, on the other hand, is not. Therefore, the quality of the approximation depends on how close to shift-invariant the summed matrix operation is. Recall that ${\mathbf {A}}$ is defined as ${\mathbf {A}} = {\mathbf {I}} - \alpha {\mathbf {W}}^{\rm T}{\mathbf {W}}$. By definition, the identity operator ${\mathbf {I}}$ is shift-invariant, so the question remains how close to shift-invariant the combined ${\mathbf {W}}^{\rm T}{\mathbf {W}}$ operation is.

Different ways of implementing the system matrix ${\mathbf {W}}$ are used in practice, each using a different discretization of the modeled tomography experiment. Depending on the computational hardware that is used (e.g., GPUs or CPUs), some implementations can be significantly more computationally efficient compared with others [17]. In most cases, reconstruction results are not significantly impacted by the specific choice of system matrix implementation [30]. It turns out, however, that how close ${\mathbf {W}}^{\rm T}{\mathbf {W}}$ is to being shift-invariant *is* significantly impacted by the choice of discretization.

Often, a single ray of the tomographic experiment is modeled as a line, with the element $w_{ij}$ of ${\mathbf {W}}$ equal to the intersection of pixel *j* with the line corresponding to ray *i*. Using this definition, the ${\mathbf {W}}^{\rm T}{\mathbf {W}}$ operation is not approximated well by a shift-invariant convolution operation. The approximation can be improved by using *supersampling*, i.e., using multiple lines per ray, and combining the results afterwards. A more accurate approximation can be obtained, however, by modeling a single ray by a *strip* with the same width as a detector pixel, and $w_{ij}$ equal to the overlap of strip *i* and pixel *j*. By using multiple strips per ray, it is possible to use supersampling with the strip model as well. Reconstruction results for several choices of discretization are shown in the “Results and discussion” section. Note that the specific choice of discretization is only important during the computation of the filter. When performing the final reconstruction with Eq. (14), all discretizations that work well for standard FBP can be used effectively.

### Even numbers of pixels

Computing the filter ${\mathbf {q}}_n$ using Eq. (12) requires setting the central pixel of image ${\mathbf {e}}_c$ to one. This presents a problem when the number of rows or columns of ${\mathbf {e}}_c$ is even, since there is no clear central pixel when there is an even number of pixels. A simple solution is to increase or decrease the number of rows or columns by one in these cases. The number of pixels in modern tomographic experiments is typically relatively large (e.g., several thousands), which makes the error made by this approximation relatively small.

### Number of iterations

The SIRT-FBP method has a single parameter that influences the reconstruction results: the number of iterations of SIRT that are approximated. A larger number of approximated iterations will increase the required computation time for computing the SIRT-FBP filter, but does not influence the required computation time for reconstruction. The properties of the reconstructed image, however, do depend on the chosen number of iterations. In the iterative SIRT method, choosing a certain number of iterations can be viewed as a type of regularization: in general, using few iterations will result in images with less noise, but less high-frequency details as well, while using many iterations results in images with more details but more noise as well. Since the SIRT-FBP method approximates the SIRT method, the effect of the number of iterations on the reconstructed image is similar. The optimal choice of this parameter depends on the geometry of the tomography experiment, the type of objects that were scanned, and the type of analysis that is performed after reconstruction. Reconstruction results of the SIRT and SIRT-FBP method are typically not very sensitive to the specific chosen number of iterations, however, and reasonable choices typically give satisfactory results in practice. In some cases, it is also possible to choose the parameter based on a stopping criterion [31].

### Low-frequency artifacts

Empirically, we find that most artifacts resulting from approximating SIRT by SIRT-FBP occur in the low frequencies of the reconstructed image. Note that a similar effect is found when discretizing the Ram-Lak filter of standard FBP [4, Fig. 3.13]. Typically, the artifacts are small enough to be invisible to a human observer. They can be further reduced if needed, however, by removing low-frequency signals from the acquired data and adding them back in the image after reconstruction. Here, we use a simple approach that we empirically found to work well for the SIRT-FBP method: we subtract from the acquired data the simulated projections of a uniform disk, which is as large as the field-of-view and centered on the rotation axis. The uniform gray value of the disk is chosen such that the zero-frequency component of each projection is minimized after the subtraction. After reconstruction, the disk can be added back to the reconstructed image. Specifically, let ${\mathbf {p}}_{C} \in {\mathbb {R}}^{N_d N_{\theta }}$ be the simulated projections of the disk with a gray value of one, let $\overline{{\mathbf {p}}} \in {\mathbb {R}}^{N_{\theta }}$ be the zero-frequency component of the acquired data along each projection angle, and let $\overline{{\mathbf {p}}_C} \in {\mathbb {R}}^{N_{\theta }}$ be defined similarly. Then, the chosen gray value of the subtracted disk is defined as

15 $$\begin{aligned} a_C = \underset{a}{{{\text {argmin}}}} \Vert \overline{{\mathbf {p}}} - a \overline{{\mathbf {p}}_C} \Vert _2. \end{aligned}$$

By performing this additional preprocessing step, the effect of the low-frequency artifacts can be minimized, as shown in the “Results and discussion” section.

### Filtering after backprojecting

Reconstructions of the SIRT-FBP method can be computed in two ways: convolving the projection data first and backprojecting afterwards [Eq. (14)], or backprojecting the projection data first and filtering afterwards [Eq. (11)]. In general, both approaches will produce similar reconstruction results, but the backproject-then-filter (BTF) approach has several disadvantages compared to the filter-then-backproject (FTB) approach. For example, the BTF approach requires a two-dimensional convolution with image ${\mathbf {q}}_n$, while the FTB approach uses multiple one-dimensional convolutions, which is computationally more efficient. More importantly, the BTF approach requires that the backprojection is performed on a grid that is significantly larger than ${\mathbf {q}}_n$, since severe edge effects will occur otherwise in the reconstructed image. Similar to standard FBP, the FTB approach does not require backprojecting on a large grid, making it significantly more efficient computationally compared with the BTF approach. A comparison of reconstruction results of the BTF and FTB approaches is given in the “Results and discussion” section.

### Using gridrec instead of FBP

In practice, the gridrec method [5] is often used to reconstruct large-scale tomographic datasets efficiently. The gridrec method can be viewed as an approximation to the standard FBP method, making it more suitable for efficient computation using standard CPUs. Importantly, the gridrec method includes a filtering operation that is identical to the filtering operation in standard FBP. Therefore, it is possible to use the filters computed by the SIRT-FBP method in gridrec as well, which enables very efficient computation of SIRT-FBP reconstructions on both workstations [32] and large-scale supercomputers. Results of the “Results and discussion” section show that SIRT-FBP reconstructions computed with gridrec are visually similar to standard SIRT-FBP reconstructions.

### Filter computation time

The most time-consuming part of computing a SIRT-FBP filter is performing the iterations of Eq. (12). In each iteration, a single forward projection and a single backprojection operation are needed, similar to the standard iterative SIRT method. The total required time for computing a single filter is therefore similar to the time it takes to compute a SIRT reconstruction for a single slice. Note, however, that the filter can be precomputed for a specific acquisition geometry (i.e., number of projections and detector column pixels, and size of the reconstruction grid). The precomputed filter can be stored and used for future SIRT-FBP reconstructions using the same acquisition geometry. Furthermore, filters for multiple numbers of iterations can be computed in a single run by storing the filter at each chosen number of iterations. Finally, a full three-dimensional tomographic reconstruction using SIRT-FBP can be computed slice-by-slice, with each slice using the same filter.

### Truncated data

In some tomographic experiments, the scanned object is larger than the field-of-view (FOV) of the scanning hardware. In these cases, the projection data are *truncated* at the edge of the detector. This truncation can lead to severe artifacts in the reconstructed images. When reconstructing the data using FBP or gridrec, truncation artifacts can be reduced by *padding* the data to a larger virtual detector [33]. Typically, the measured values at the edges of the detector are used as virtual measurements in the padded regions, ensuring that there is no large jump in the values at the detector edges. Note that it is not possible to apply this approach to iterative methods like SIRT. Instead, a common approach for iterative methods is to use a reconstruction grid that is larger than the FOV, which can increase the required computation time significantly. In the SIRT-FBP method, on the other hand, it *is* possible to use the padding approach, since it can be viewed as standard FBP with special filters. Therefore, it is possible to compute approximated iterative reconstructions of truncated data very efficiently using the SIRT-FBP method. Note that in these cases, the computation of the SIRT-FBP filter has to be performed on a grid that is larger than the FOV, but the reconstruction itself only uses a backprojection as large as the FOV.

## Results and discussion

In this section, we will discuss results of applying the SIRT-FBP method on large-scale tomographic data, and the implications on large-scale tomographic reconstruction in practice. First, we will discuss the impact of the various implementation details that were discussed in the “Implementation details” section. Afterwards, we show three examples of applying the SIRT-FBP method on large-scale real-world tomographic data. The computer code that computes SIRT-FBP filters was implemented using the ASTRA toolbox [34], and will be made available under an open-source license. Note that, after filter computation, existing efficient implementations of the FBP method and gridrec method can be used to compute SIRT-FBP reconstructions. In all reconstruction results presented in this paper, the number of rows and columns of the reconstruction grid is identical to the number of detector column pixels.

### Implementation details

**Fig. 1:**
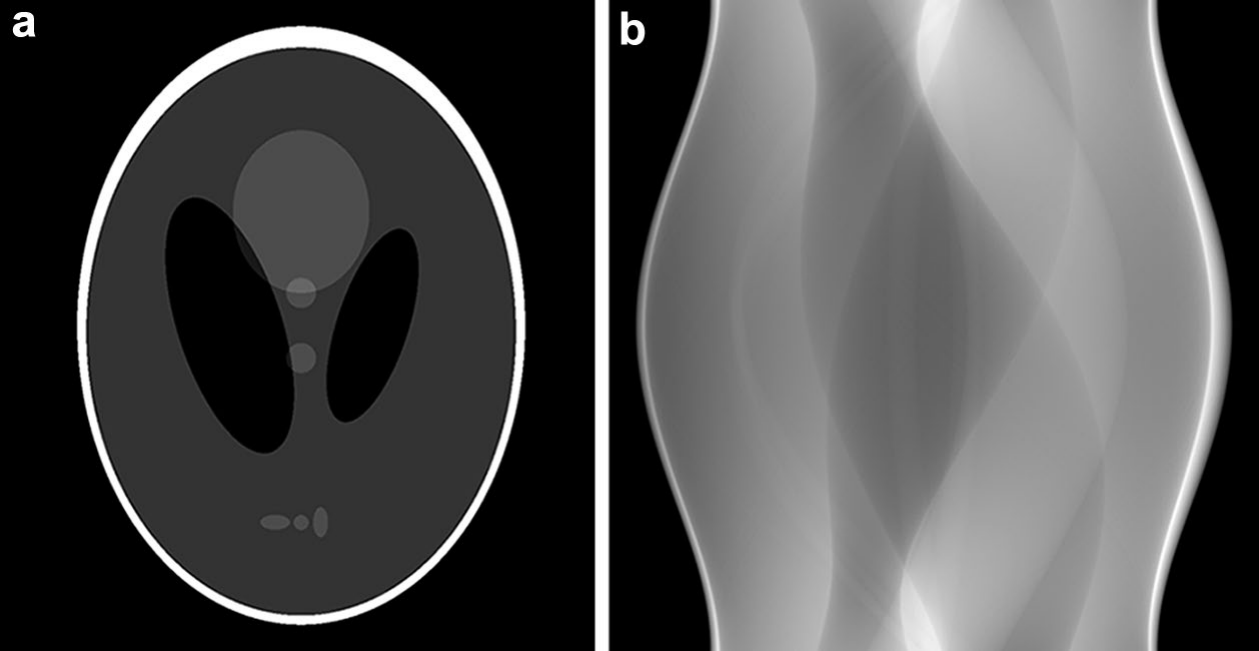
**a** Shepp–Logan head phantom and **b** its projections for $\theta \in [0,\pi )$

To demonstrate the impact of some of the implementation details that were discussed in the “Implementation details” section, we will use reconstructions of simulated projections of the Shepp–Logan head phantom (Fig. 1). In most cases, we use 256 projections with 1024 detector pixels per projection, and reconstruct on a $1024\times 1024$ reconstruction grid.

**Fig. 2:**
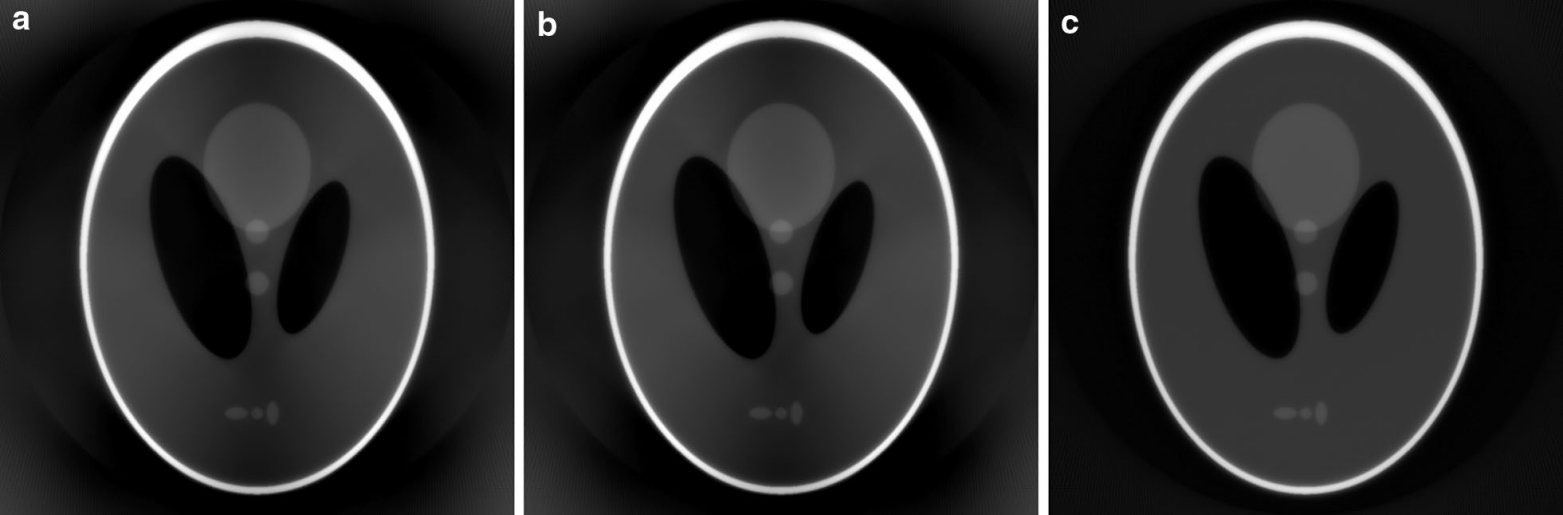
Reconstructions of the Shepp–Logan head phantom with the SIRT-FBP method, using different discretizations of the system matrix during filter computation. In **a** the Joseph kernel is used, in **b** rays are modeled by a line, and in **c** rays are modeled by a strip with the same width as a detector pixel

**Fig. 3:**
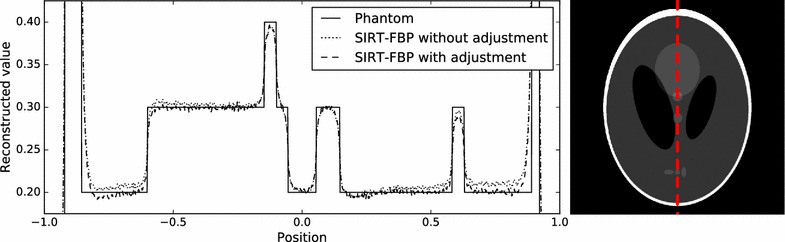
Line profile of SIRT-FBP reconstructions of the Shepp–Logan head phantom, with and without low-frequency artifact adjustment. Values are shown for the *vertical line* in the *horizontal center* of the reconstruction grid, which is indicated by the *dashed line in the right image*

**Fig. 4:**
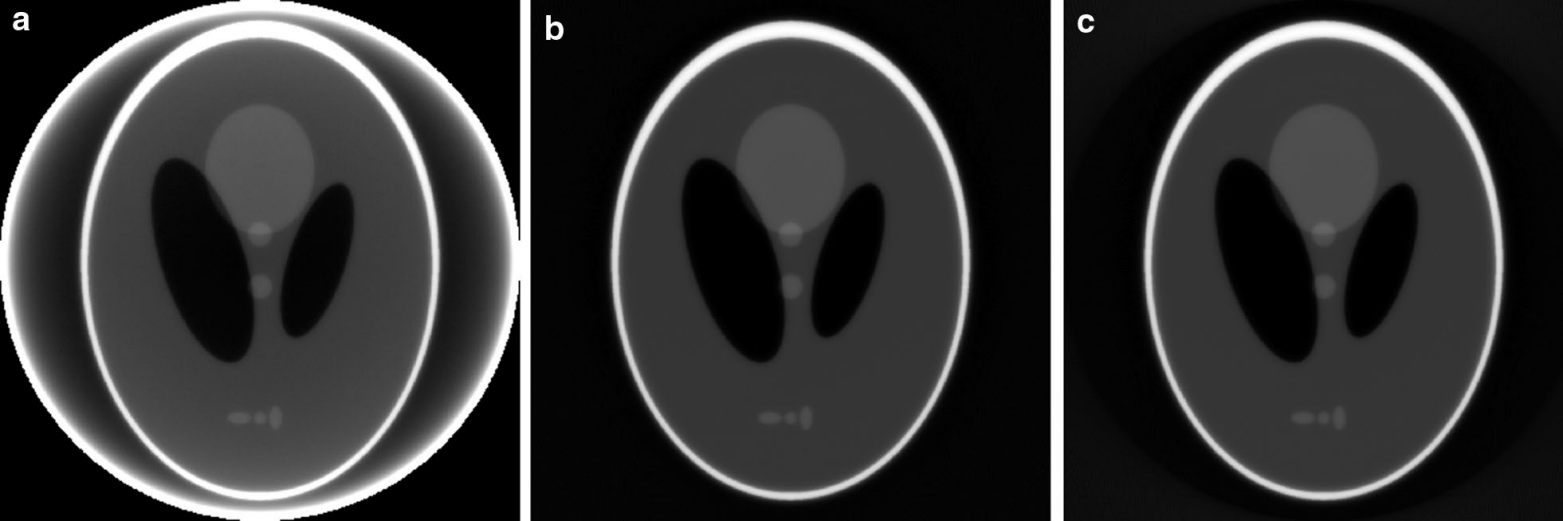
SIRT-FBP reconstructions of the Shepp–Logan head phantom using **a**, **b** the BTF approach and **c** the FTB approach. In **a** and **c** the backprojection operation is performed using a grid as large as the field-of-view, while in **b** a grid that is four times larger than the field-of-view is used

**Fig. 5:**
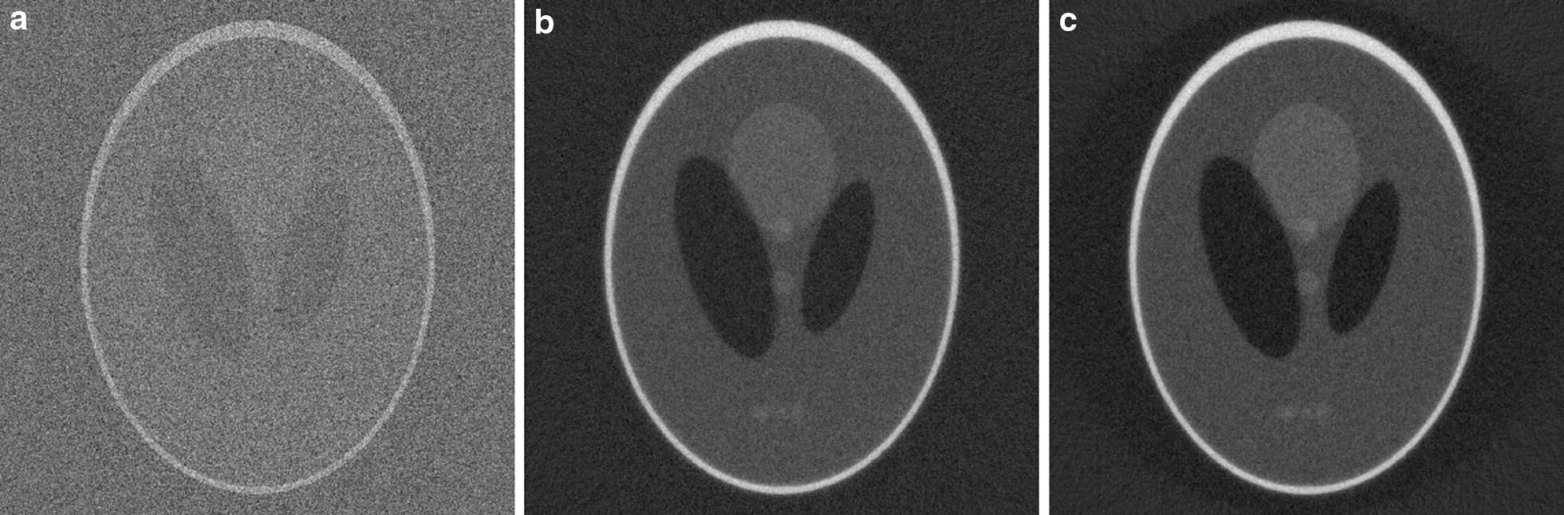
Reconstructions of the Shepp–Logan head phantom from noisy projection data, using **a** standard gridrec with the Shepp–Logan filter, **b** gridrec with the SIRT-FBP filter, and **c** FBP with the SIRT-FBP filter

**Fig. 6:**
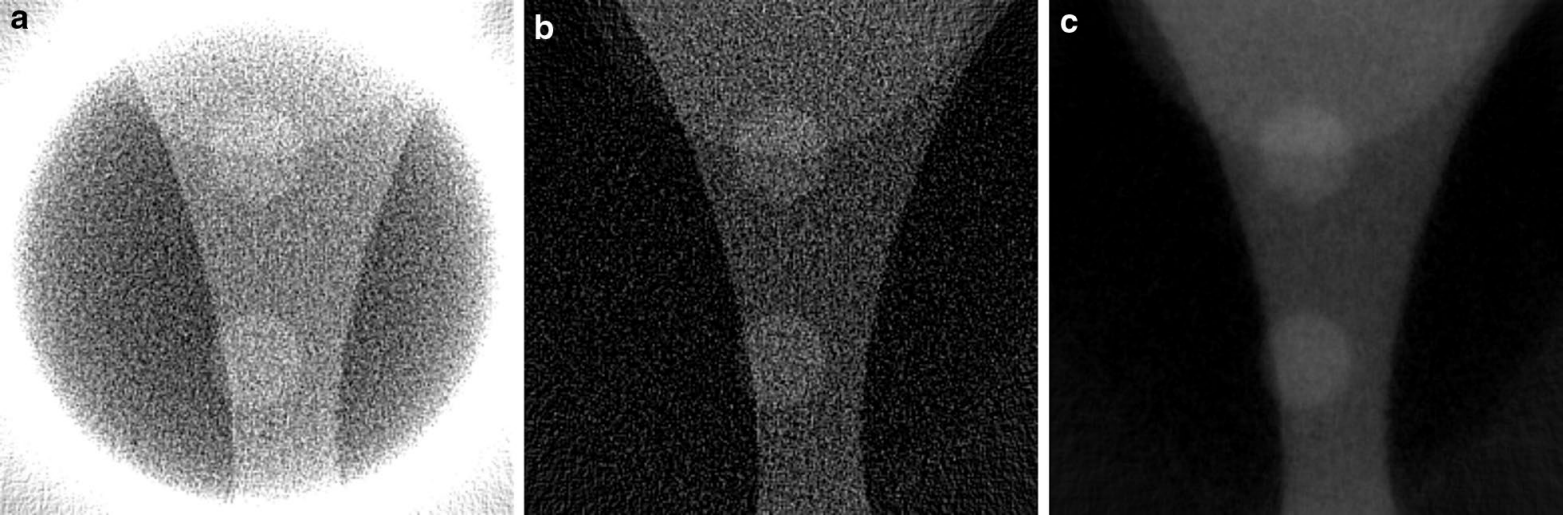
Reconstructions of the Shepp–Logan head phantom from noisy projection data truncated to the central 256 pixels, using **a** standard FBP without padding, **b** standard FBP with padding, and **c** SIRT-FBP with padding

A comparison of reconstruction results using different system matrix discretizations is given in Fig. 2, showing that artifacts occur when the system matrix discretization is not approximately shift-invariant. In Fig. 3, a comparison is made between reconstruction results of the SIRT-FBP method both with and without the low-frequency adjustment. The results show that the low-frequency adjustment is able to minimize the minor low-frequency artifacts that occur with standard SIRT-FBP. Since the effect of the low-frequency artifacts is typically small, we did not apply the adjustment in the other examples of this paper. In Fig. 4, a comparison is made between reconstructions with the backproject-then-filter (BTF) and filter-then-backproject (FTB) approaches, both with and without backprojecting on a larger grid. The results show that the reconstructed image contains severe artifacts when using the BTF approach without a large grid, while reconstructions of the BTF approach with a large grid and the FTB approach without a large grid are visually similar. In Fig. 5, a comparison is made between reconstructions computed with SIRT-FBP using FBP, SIRT-FBP using gridrec, and standard gridrec. Note that both SIRT-FBP reconstructions are visually similar, showing that it is possible to accurately approximate the SIRT-FBP method with gridrec. Finally, a comparison of reconstruction results for truncated data with various reconstruction methods is shown in Fig. 6.

**Fig. 7:**
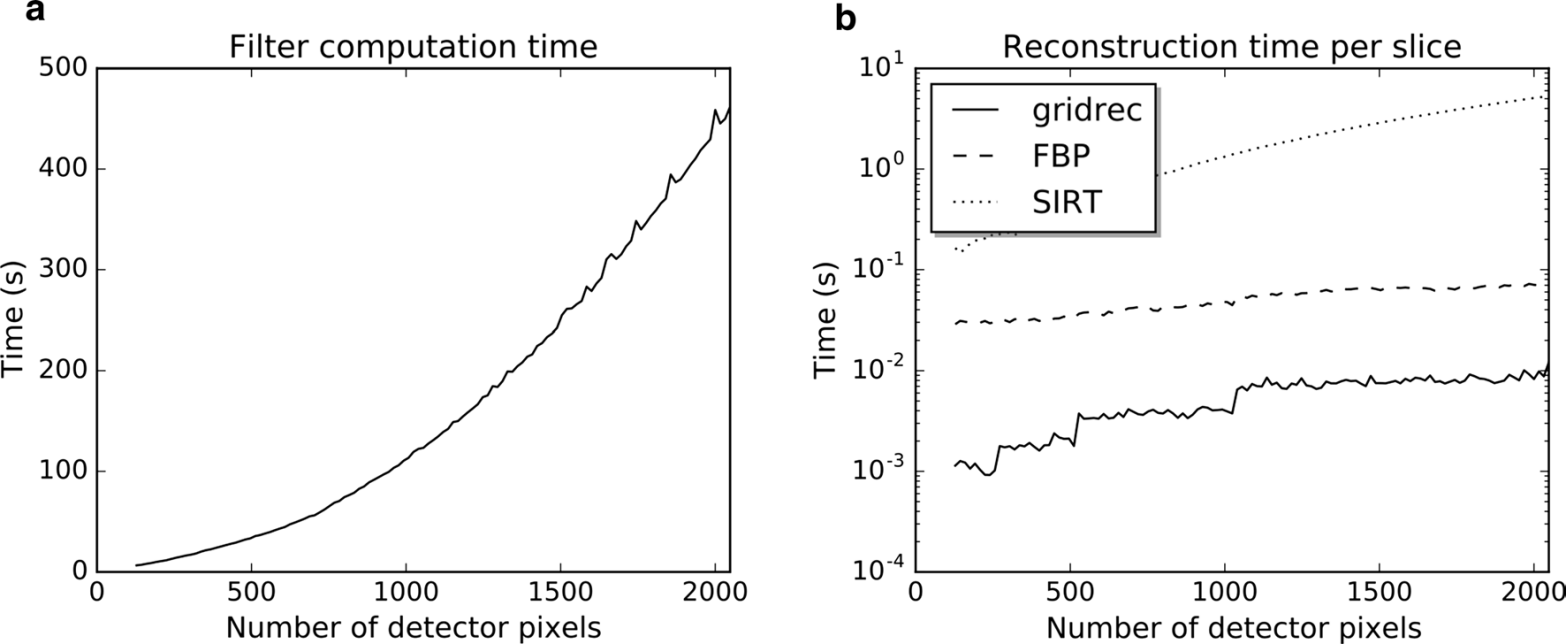
Computation times for 512 projections and various numbers of detector pixels. In each case, the number of rows and columns in the reconstruction grid is identical to the number of detector pixels per projection. In **a**, the required time for computing SIRT-FBP filters is shown for 100 iterations using 16 CPU cores, and in **b** reconstruction times are shown for 100 iterations of SIRT, and the SIRT-FBP method using both FBP and gridrec. The FBP reconstructions are computed using a single GPU, and the gridrec reconstructions are computed using 16 CPU cores

Measured times for both computing SIRT-FBP filters and reconstructing with SIRT-FBP are shown in Fig. 7, including a comparison with the reconstruction time of standard SIRT. All computations were performed on a workstation with 16 CPU cores (Intel Xeon E5-2630) and a single NVidia Tesla K80 GPU. Note that even for large detector sizes, computing the SIRT-FBP filter takes <10 min. Furthermore, the results of Fig. 7b show that it takes significantly less time to reconstruct images using the SIRT-FBP method compared with the iterative SIRT method. This reduction in reconstruction time can have significant impact in practice: on this workstation, for example, reconstructing a full $2048\times 2048\times 2048$ volume from 512 projections would take more than 3 h using SIRT with one GPU, whereas reconstructing the same data with the SIRT-FBP method would take roughly 146 s using FBP with one GPU, and roughly 25 s using gridrec. Note that the required computation time of FBP and iterative methods generally scales linearly with the number of acquired projections. In the gridrec method, on the other hand, the number of projections has a relatively small influence on the computation time, since the most time-consuming operations of gridrec are its 2D Fourier transforms, which do not depend on the number of acquired projections.

### Experimental data

In this section, we show reconstruction results of the SIRT-FBP method for three examples of large-scale real-world tomographic data. The results are compared with standard gridrec reconstructions using various popular filters and with the iterative SIRT method. The data were acquired with a transmission X-ray microscope (TXM) setup at the 32-ID beamline of Argonne National Laboratory’s Advanced Photon Source [35]. After acquisition, the projections were processed with TomoPy [32] and reconstructed with either TomoPy (all gridrec reconstructions) or the ASTRA toolbox [34] (all SIRT-FBP and SIRT reconstructions), using the recent integration of both toolboxes [18].

#### Diamond anvil cell

The first dataset is an example of a common in situ setup in which a sample was put under increasing pressures in a diamond anvil cell. The frame of the anvil cell blocks the incoming beam for many projection angles, making it impossible to acquire projections for the entire 180$^\circ$ range. These *limited-angle* problems are common in electron tomography [36] and make it difficult to obtain accurate reconstructions, especially using direct reconstruction methods [37]. In the dataset we discuss here, projections of $2160\times 2560$ pixels were acquired in 0.5$^\circ$ intervals over a 137$^\circ$ range, resulting in 273 projections.

**Fig. 8:**
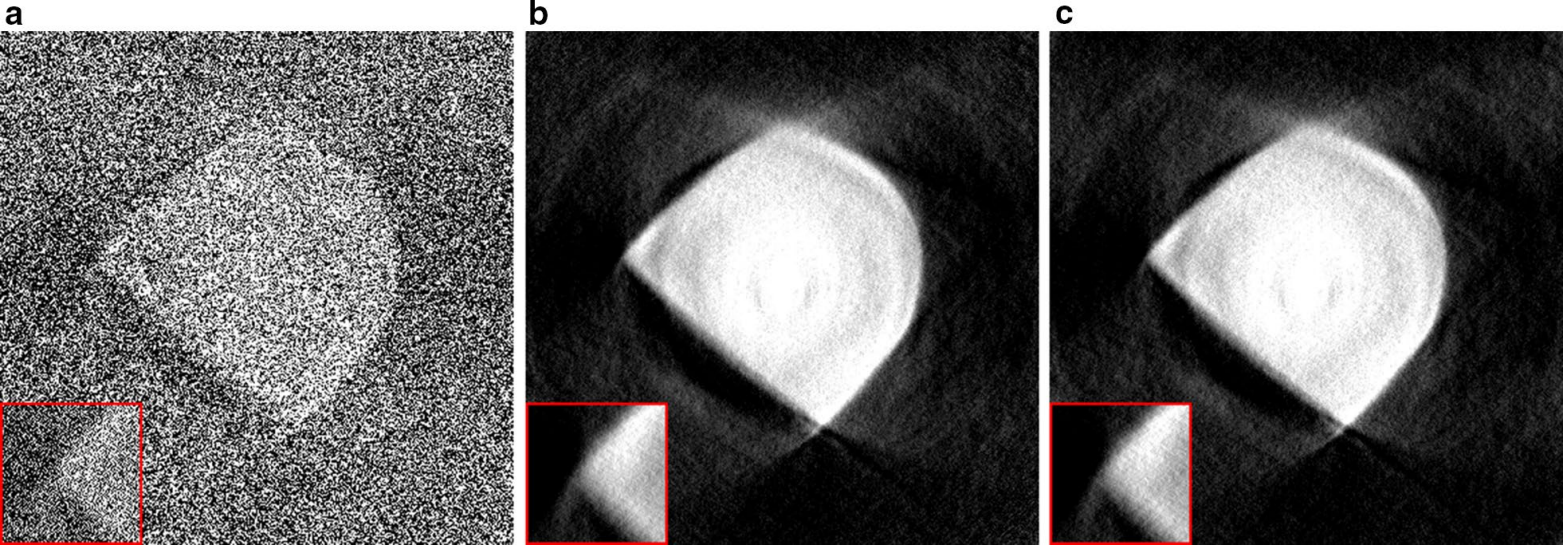
Reconstructions of a diamond anvil cell experiment with missing projections, using: **a** the standard gridrec reconstruction with the Shepp–Logan filter, **b** the standard iterative SIRT method with 100 iterations, and **c** the proposed SIRT-FBP method with 100 approximated iterations

In Fig. 8, reconstruction results are shown of a single slice using the gridrec method, the iterative SIRT method, and the SIRT-FBP method. Note that the gridrec reconstruction contains significantly more noise compared with the other reconstructions. Furthermore, the results show that the SIRT-FBP reconstruction is visually very similar to the iterative SIRT reconstruction. However, computing a single slice of the SIRT-FBP reconstruction takes 75 ms using a NVidia Tesla K80 GPU, while computing a single slice of the SIRT reconstruction takes 4.9 s using the same GPU.

**Fig. 9:**
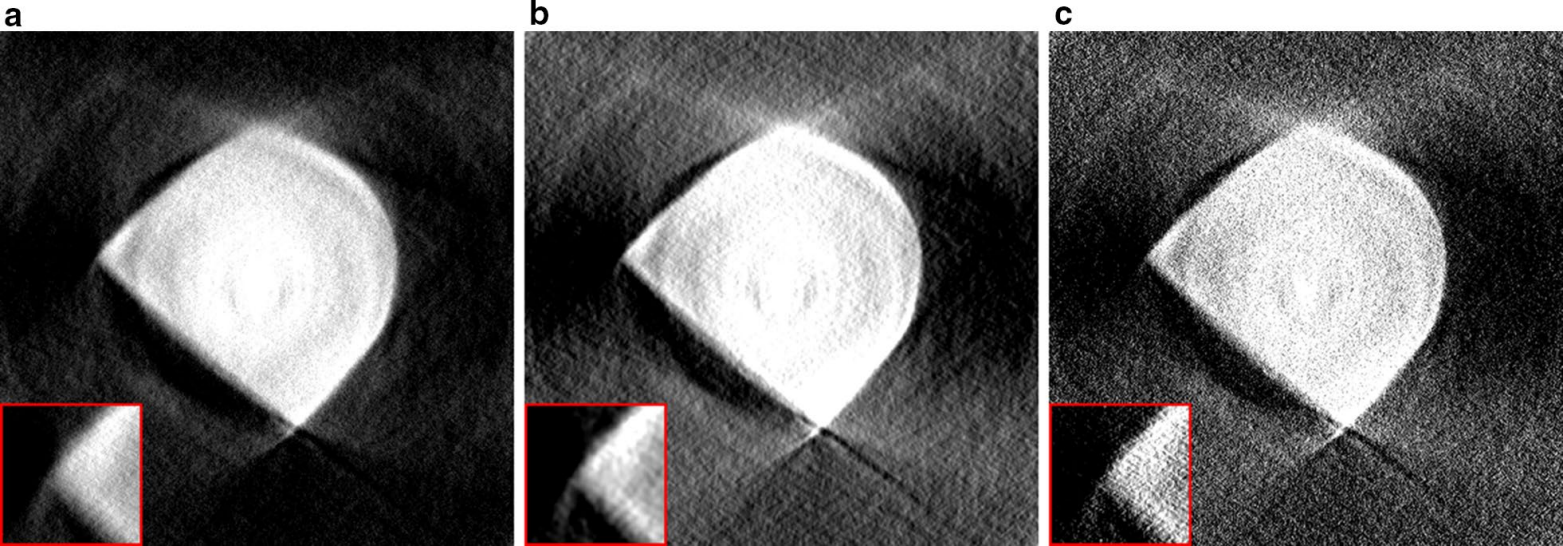
Reconstructions of a diamond anvil cell experiment with missing projections, using: **a** the proposed SIRT-FBP method with 100 approximated iterations, **b** standard gridrec with the Shepp–Logan filter and additional gaussian filtering afterwards, and **c** standard gridrec with the Parzen filter

A common approach for reducing noise artifacts in direct reconstructions is to either change the convolution filter, or to apply additional filtering to the image after reconstruction. Note that additional filtering usually requires choosing parameters that have a large influence on the final image quality, and can increase the required processing time for large-scale data significantly. In Fig. 9, the SIRT-FBP reconstruction is compared with reconstructions computed using gridrec with the Shepp–Logan filter and additional Gaussian filtering, and using gridrec with the Parzen filter, which is often used in problems with low signal-to-noise ratios. The results show that although both approaches are able to reduce noise artifacts compared with standard gridrec using the Shepp–Logan filter (Fig. 8a), the SIRT-FBP reconstruction contains less artifacts. Furthermore, the reconstructions using additional filtering and the Parzen filter contain significantly more limited-angle artifacts compared with the SIRT-FBP reconstruction. A possible reason for this is the fact that the SIRT-FBP filter is computed specifically for the limited-angle geometry of the actual experiment, while the standard filters and additional filtering steps do not take the limited-angle geometry into account.

#### Mouse cortex

**Fig. 10:**
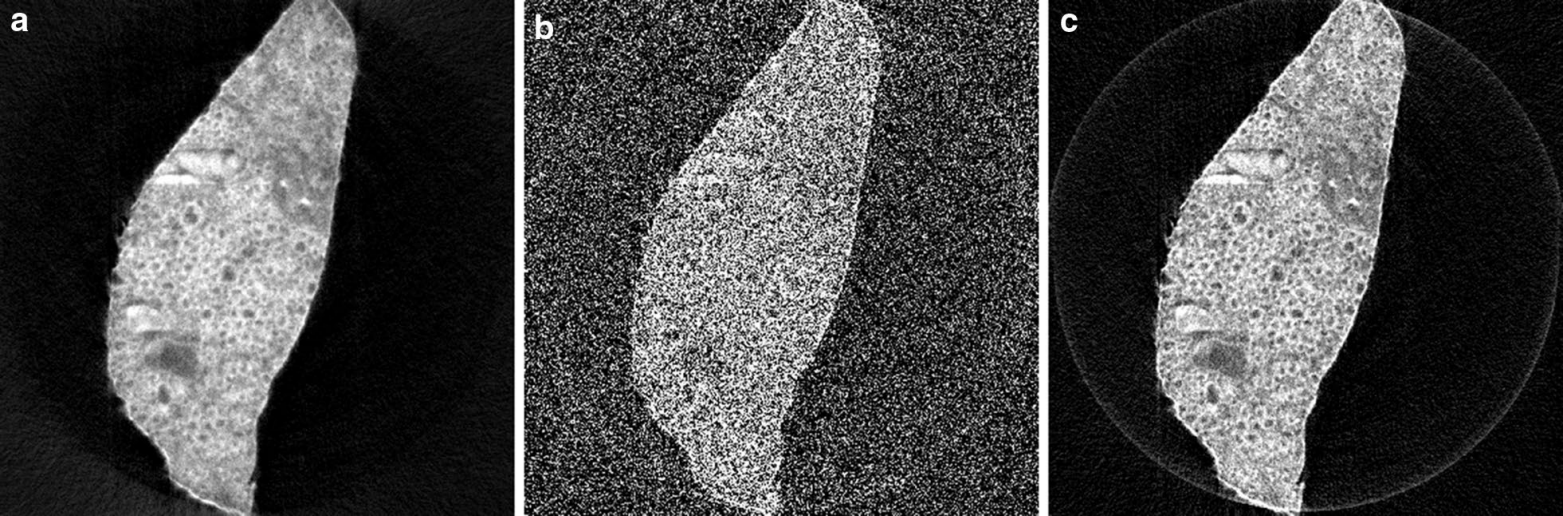
Reconstructions of a mouse cortex, using: **a** the proposed SIRT-FBP method with 100 approximated iterations, **b** standard gridrec with the Shepp–Logan filter, and **c** standard gridrec with the Parzen filter

The second scanned object consists of large bundle of myelinated axons in the cortex of a mouse. Samples were prepared for routine electron microscopy (i.e., fixed with aldehydes, stained with osmium, dehydrated, and embedded in plastic). Data were acquired for 1501 projections over 180$^\circ$ using a detector with $2048 \times 2448$ pixels, binned to 1124 detector pixels. In Fig. 10, reconstructions of a single slice are shown for the SIRT-FBP method and gridrec using the Shepp–Logan filter and the Parzen filter. Note that the gridrec reconstruction using the Shepp–Logan filter contains significantly more noise artifacts compared with the other two reconstructions. Specifically, the myelin rings of the axons are clearly visible in the reconstructions using SIRT-FBP and the Parzen filter, but not in the reconstruction using the Shepp–Logan filter, due to severe noise artifacts. The reconstructions of the SIRT-FBP method and gridrec using the Parzen filter are visually similar, with the SIRT-FBP reconstruction containing slightly less noise artifacts, but having a slightly lower resolution as well. If required for the analysis, however, it is possible to increase the number of approximated iterations in the SIRT-FBP method to increase the resolution of the reconstruction.

#### Li–O$_{2}$ battery cathode

The third scanned object is a Li–O$_{2}$ battery cathode consisting of Timcal superP carbon (+10 wt% PVdF-HFP), in which Li–O$_{2}$ particles have been formed during electrochemical discharge. For this new generation of battery, obtaining accurate three-dimensional information about the material morphology is crucial to get a better understanding of the formation mechanism and behavior of the Li–O$_{2}$ depositions. The sample was principally made of carbon features which have very weak absorption at 8 keV, i.e., in the hard X-ray regime. Therefore, it was analyzed with the Zernike phase contrast approach involving a phase ring that shifts the phase of the direct beam by $\pi /2$ to transform the phase shift generated by the sample into a detectable amplitude signal, enhancing contrasts.

**Fig. 11:**
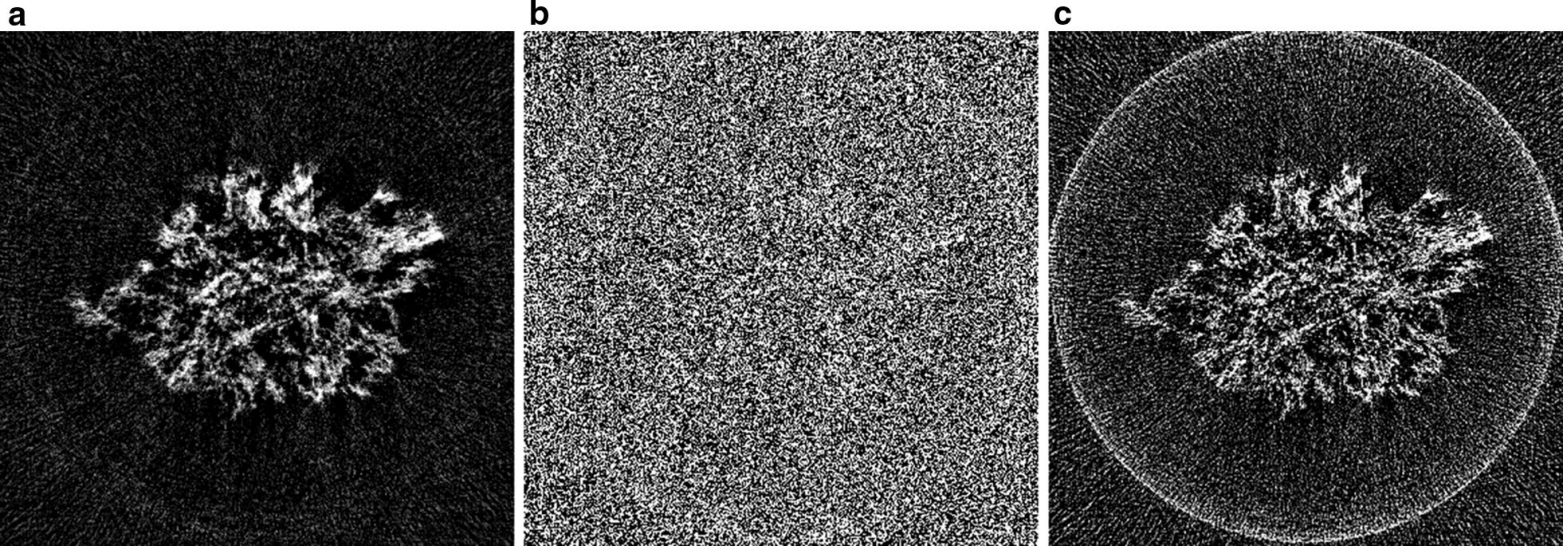
Reconstructions of a Li–O$_{2}$ battery cathode, using: **a** the proposed SIRT-FBP method with 200 approximated iterations, **b** standard gridrec with the Shepp–Logan filter, and **c** standard gridrec with the Parzen filter

In Fig. 11, reconstructions are shown of a single slice of the cathode, reconstructed from 375 projections over 180$^\circ$ with 1124 detector column pixels, using the SIRT-FBP method and gridrec with the Shepp–Logan filter and the Parzen filter. The results show that the SIRT-FBP reconstruction contains significantly less noise artifacts than the gridrec reconstructions, especially the reconstruction using the Shepp–Logan filter. Specifically, the morphology of the cathode is most clearly visible in the SIRT-FBP reconstruction, enabling more accurate analysis compared with the other reconstructions.

## Conclusions

In this paper, we have shown the application of the computationally efficient SIRT-FBP method to large-scale real-world tomographic data. The SIRT-FBP method approximates the iterative SIRT method by FBP with computed angle-dependent filters. Reconstructions computed by the SIRT-FBP method are visually very similar to reconstructions computed by the SIRT method, but the required computation time for SIRT-FBP is identical to that of standard FBP and gridrec. SIRT-FBP filters can be precomputed for a certain experimental setup by an iterative algorithm similar to the SIRT method, and experiments using an identical setup can use the same filter without recomputation. The required computation time for computing a single filter is similar to the computation time of a SIRT reconstruction for a single slice.

We have specifically focused on discussing important implementation details related to problems that occur when applying a mathematical algorithm to real-world data. For example, we explained how to minimize reconstruction artifacts by using shift-invariant discretizations of the system matrix, and how to compute filters and reconstructions for large-scale data in a computationally efficient way. Furthermore, we discussed the influence of the algorithm parameters on reconstruction quality and computation time. Reconstruction results of the SIRT-FBP method were shown for three different real-world experimental datasets acquired at the 32-ID beamline of the Advanced Photon Source. The reconstructed images were compared with those of various popular reconstruction methods, showing that the SIRT-FBP method can improve reconstruction results for large-scale real-world data without increasing the required computation time.
